# Breast Cancer Prediction Using Fine Needle Aspiration Features and Upsampling with Supervised Machine Learning

**DOI:** 10.3390/cancers15030681

**Published:** 2023-01-22

**Authors:** Rahman Shafique, Furqan Rustam, Gyu Sang Choi, Isabel de la Torre Díez, Arif Mahmood, Vivian Lipari, Carmen Lili Rodríguez Velasco, Imran Ashraf

**Affiliations:** 1 Department of Information and Communication Engineering, Yeungnam University, Gyeongsan 38541, Republic of Korea; 2 School of Computer Science, University College Dublin, D04 V1W8 Dublin, Ireland; 3 Department of Signal Theory and Communications and Telematic Engineering, University of Valladolid, Paseo de Belén 15, 47011 Valladolid, Spain; 4 Department of Computer Science & Information Technology, The Islamia University of Bahawalpur, Bahawalpur 63100, Punjab, Pakistan; 5 Research Group on Foods, Nutritional Biochemistry and Health, Universidad Europea del Atlántico, Isabel Torres 21, 39011 Santander, Spain; 6 Department of Project Management, Universidad Internacional Iberoamericana, Campeche 24560, Mexico; 7 Fundación Universitaria Internacional de Colombia Bogotá, Bogotá 11001, Colombia; 8 Department of Project Management, Universidad Internacional Iberoamericana Arecibo, Arecibo, PR 00613, USA; 9 Project Management, Universidade Internacional do Cuanza, Cuito EN250, Bié, Angola

**Keywords:** breast cancer prediction, feature selection, fine-needle aspiration features, principal component analysis, singular value decomposition, deep learning

## Abstract

**Simple Summary:**

Breast cancer is prevalent in women and the second leading cause of death. Conventional breast cancer detection methods require several laboratory tests and medical experts. Automated breast cancer detection is thus very important for timely treatment. This study explores the influence of various feature selection technique to increase the performance of machine learning methods for breast cancer detection. Experimental results shows that use of appropriate features tend to show highly accurate prediction.

**Abstract:**

Breast cancer is one of the most common invasive cancers in women and it continues to be a worldwide medical problem since the number of cases has significantly increased over the past decade. Breast cancer is the second leading cause of death from cancer in women. The early detection of breast cancer can save human life but the traditional approach for detecting breast cancer disease needs various laboratory tests involving medical experts. To reduce human error and speed up breast cancer detection, an automatic system is required that would perform the diagnosis accurately and timely. Despite the research efforts for automated systems for cancer detection, a wide gap exists between the desired and provided accuracy of current approaches. To overcome this issue, this research proposes an approach for breast cancer prediction by selecting the best fine needle aspiration features. To enhance the prediction accuracy, several feature selection techniques are applied to analyze their efficacy, such as principal component analysis, singular vector decomposition, and chi-square (Chi2). Extensive experiments are performed with different features and different set sizes of features to investigate the optimal feature set. Additionally, the influence of imbalanced and balanced data using the SMOTE approach is investigated. Six classifiers including random forest, support vector machine, gradient boosting machine, logistic regression, multilayer perceptron, and K-nearest neighbors (KNN) are tuned to achieve increased classification accuracy. Results indicate that KNN outperforms all other classifiers on the used dataset with 20 features using SVD and with the 15 most important features using a PCA with a 100% accuracy score.

## 1. Introduction

Cancer has been among the top five diseases in women over many years; globally, breast and cervical cancer have been regarded as the common cause of death from cancer between the age of 15 to 65 years among women [1]. With nonmelanoma of the skin excluded, breast cancer is the most often diagnosed cancer for women in the US. Compared to lung cancer, it is the second most common cancer among women overall, but it is the most common among Black and Hispanic women [2]. Breast cancer has been diagnosed in both men and women, but the ratio of women is higher than in men. According to the statistical report of the world cancer research fund (WCRF), approximately two million new cases were registered for breast cancer in 2018 [3]. Asian countries especially, such as Pakistan and India have the highest number of patients with breast cancer. According to a report, approximately 178,388 new cases were registered in Pakistan in the year 2020 [4]. The highest number of reported deaths in one calendar year is for 2020 when 685,000 people died worldwide as a result of breast cancer and 2.3 million women were affected. The most common disease in the globe as of the end of 2020 was breast cancer, which had been diagnosed in 7.8 million women in the previous five years [5].

Several risk factors are associated with breast cancer such as female sex, obesity, alcohol use, hormone therapy during menopause, no or less physical activity, having children later in life or not at all [6]. Several kinds of tumors can appear in various breast regions and are broadly categorized as noninvasive and invasive. Noninvasive breast cancer cells stay in the ducts and do not infiltrate the breast’s fatty and connective tissues. The majority (90%) of noninvasive breast cancer cases are caused by ductal carcinoma in situ (DCIS). LCIS, a less frequent condition, is thought to increase the chance of developing breast cancer. Invasive breast cancer cells infect the breast’s surrounding fatty and connective tissues by penetrating the duct and lobular walls. Without metastasis (spreading) to the lymph nodes or other organs, cancer can be invasive. Thus, its timely prediction would make the treatment possible at earlier stages and could save countless lives.

Early prediction of breast cancer is very important, but the conventional diagnosis process is long and involves several medical tests once recommended by a medical expert. It requires both time and money and often the prediction varies from one medical expert to another. Therefore, an automated diagnosis system is highly desired to predict breast cancer efficiently, timely, and accurately. Many traditional methods are used to diagnose breast cancer such as mammography, ultrasound, and magnetic resonance imaging (MRI) [7]. Predominantly, mammography and ultrasound are used to find the area affected by cancer. These methods use screening platforms where radiology images (X-ray) of the breast are taken and then analyzed by medical experts for diagnosis.

Another approach that can accurately identify breast cancer is fine-needle aspiration (FNA), a kind of biopsy, to collect tissue and fluid samples from solid or cystic breast lesions. It is one of the several methods for identifying breast lumps that are not removed formally. Many research works used FNA features for various diseases of the breast using datasets that comprise visually measured atomic features which are explained in [8]. For this purpose, various attributes of FNAs such as texture, concaveness, smoothness, etc., are used with machine and deep learning approaches. For example, the authors in [8] utilized FNA features to predict breast cancer by using various machine learning approaches. The use of a support vector machine (SVM) is reported to achieve 92.7% accuracy for breast cancer prediction using FNA features. Similarly, the study [9] diagnosed breast cancer by a new approach called RS-SVM (rough set–SVM) to remove redundant attributes and improve accuracy. Despite previously presented diagnosis approaches, the desired prediction accuracy and the achieved prediction accuracy do not agree. This research aims to increase breast cancer prediction accuracy by analyzing various feature extraction approaches for their efficacy. Additionally, the role of the size of various feature sets is extensively investigated to find the optimal feature set for higher accuracy. In brief, this study makes the following contributions:

- An automated approach for breast cancer prediction is presented that utilizes fine needle aspiration features. Based on FNA, patients are categorized into benign and malignant.
- Various feature selection techniques such as principal component analysis (PCA), singular value decomposition (SVD), and chi-square (Chi2), are analyzed for their efficacy to select the best features from the dataset containing FNA features. Moreover, the impact of different sizes of feature vectors on the prediction accuracy is extensively investigated during several experiments.
- In addition to selecting important features, the impact of primary and derived features is investigated for the breast cancer detection problem where several features are derived from the primary features to increase the classification accuracy.
- Several machine learning algorithms are used for breast cancer prediction including random forest (RF), SVM, gradient boosting machine (GBM), logistic regression (LG), and k-nearest neighbors (KNN). Their performance is examined with various feature selection techniques, as well as various feature vectors for increased accuracy.
- Several experiments are performed to investigate whether the addition of more features is important or fewer features with high importance. Moreover, the performance of the proposed approach is compared with several state-of-the-art approaches.

The rest of the paper is organized as follows. Section 2 discusses the research works that are closely related to the current study. Section 3 gives a brief overview of the dataset, feature selection techniques, the machine learning algorithms used in this study, as well as the proposed methodology. Results are presented in Section 4 while the conclusion is given in Section 5.

## 2. Related Work

Cancer, especially breast cancer, has been one of the leading causes of death in women over the past few years. Several research works have been presented that use machine learning algorithms to diagnose breast cancer at various levels. These works can be grouped into two categories regarding the use of classifiers: machine learning classifiers and deep learning classifiers. Machine learning classifiers include traditional classifiers such as SVM, RF, logistic regression, etc., while the deep learning approaches focus on using neural networks including long short-term memory, gated recurrent unit, convolutional neural network, etc.

For example, the study [10] provided an analysis of various machine learning and deep learning algorithms for breast cancer prediction. Deep learning algorithms such as multilayer perceptron and neural networks (NN) with backpropagation gave the best accuracy of 99.28%. Similarly, machine learning algorithms such as SVMs gave an accuracy of 98.0%. In the same way, the authors in [11] used the relevance vector machine (RVM) for breast cancer detection. Experiments were performed for various types of cancers such as ovarian cancer, optical cancer, breast cancer, etc., where the RVM showed good performance for the detection of ovarian and optical cancers.

Another study [12] used an ensemble approach for breast cancer detection where various algorithms were used including C4.5, C5, CART, CHAID, SLIQ, SPRINT, and ScalParc. These classifiers were selected based on their best performance for various healthcare decision-support functions. The proposed approach was a hybrid solution where feature selection and bagging technique was adopted. Three breast cancer datasets were tested such as “breast cancer”, “Wisconsin breast cancer dataset (WBCD) original”, and “WBCD diagnostic” for evaluating the performance of the proposed approach. The achieved accuracy with the proposed approach was 74.47%, 97.85%, and 95.5%, respectively, for the given datasets. The study [13] used three different classifiers from the WEKA software for the classification of breast cancer. These techniques included a sequential minimal optimization (SMO), a k-nearest neighbors classifier (IBK), and a best first tree (BF). Results indicated that a better accuracy of 96.2% could be achieved using SMO for breast cancer detection.

Many research works adopt deep learning approaches for breast cancer detection. For example, the study [14] used neural networks for the classification of breast cancer. Multiple statistical neural network structures including a self-organizing map (SOM), a radial basis function network (RBF), a general regression neural network (GRNN), and a probabilistic neural network (PNN) were tested on the WBCD and NHBCD datasets. The PCA technique was also used to reduce the dimension of the data and find the best features. An RBF and PNN were proven as the best classifiers in the training set, and for the test set, a PNN gave the best classification accuracy. The overall results showed that the most suitable neural network model for classifying WBCD and NHBCD data was the PNN. This work also indicated that statistical neural networks could be effectively used for breast cancer diagnosis to help the healthcare industry.

Similarly, the authors leveraged an artificial neural network for breast cancer detection in [15]. Experiments were conducted using two different breast cancer datasets with nuclear morphometric features. Results suggested that the ANN could successfully predict recurrence probability. A comparative analysis of traditional machine learning classifiers was performed in [16] on a breast cancer dataset. The study used an SVM, naive Bayes classifier, and ANN for this purpose. Accuracy, sensitivity, and specificity results showed that the SVM performed better with an accuracy of 97.67% on the WBCD and the “opinion breast cancer problem”.

The authors proposed a hybrid method for the diagnosis of breast cancer by using various machine learning techniques in [17]. This study combined a fuzzy artificial immune system with a k-nearest neighbors classifier and evaluated its performance on the WBCD dataset. The best accuracy (91.4%) was given by the purposed hybrid model with a 10-fold cross-validation. The study [18] presented a novel approach to breast cancer diagnosis. An artificial neural network was evolved into an optimal architecture. For this, a genetically optimized neural network model (GONN) was used which was based on genetic programming. The GONN was compared with a BPNN and Koza’s models. The maximum accuracy of 99.63% was achieved using the GONN. Similarly, a new model for the classification of breast cancer was introduced in [19]. The model was based on the naïve Bayes theorem and proved to be more accurate than traditional machine learning classifiers.

Table 1 shows the summary of the research works discussed in this section. Despite the reported breast cancer detection accuracy, these works lack several aspects. First, the majority of the research works focus on tuning the machine or deep learning hyperparameters to improve the classification performance of the models. This approach is appropriate for one dataset, however, changing the dataset will change the classification results. Secondly, feature selection which is very important for attaining accuracy and precision is not extensively studied. Selecting important features helps to increase the classification accuracy on multiple datasets and the generalization of the results. To this end, this research primarily focuses on the selection of important features to increase breast cancer detection accuracy.

## 3. Material and Methods

### 3.1. Dataset Description

The dataset used for the experiments was taken from Kaggle and is available at [20]. The dataset contained two types of features i.e., categorical and numerical. The values of the features were taken from a process called a fine-needle aspiration (FNA) [21]. In an FNA, a needle is injected into the abnormal body mass or tissue, which is later analyzed for various indicators. The dataset contained 659 records with each record having 30 features and 2 target classes “benign” and “malignant”. Each feature had a real value which represented an attribute to decide whether the person was healthy or a patient. The features were calculated using the data from the tissue extracted from the body of the person using the FNA procedure. The selected dataset had three values for each attribute including mean, standard error, and maximum value. Table 2 shows the name of various attributes and associated values.

Ten attributes were selected from the dataset which had real values. The dataset had two classes “Benign” and “Malignant” and the distribution of records for each class is shown in Table 3.

**Figure 1:**
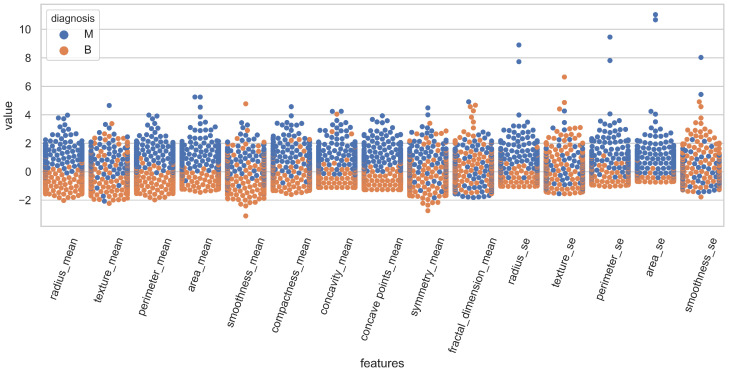
Representation of attributes 1–15 with respect to classes.

**Figure 2:**
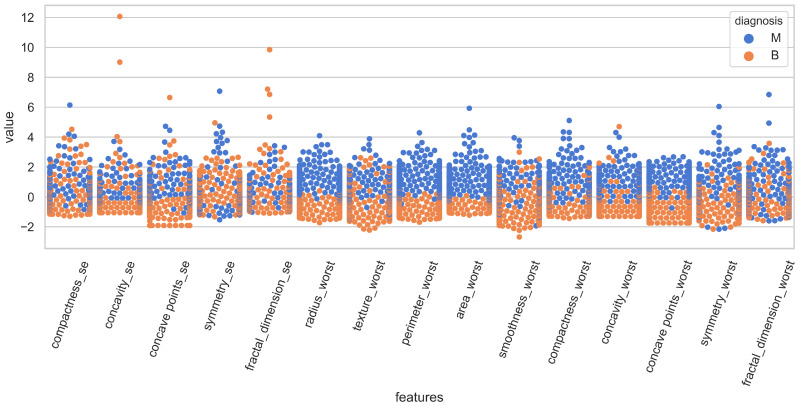
Representation of attributes 15–30 with respect to classes.

The distribution of the features for both classes is illustrated in Figure 1 using swarm plots. For a clear illustration, fifteen features are displayed in Figure 1 and Figure 2. Figure 1 and Figure 2 show the variance of various features regarding the target classes.

For example, the value of “smoothness_se” in Figure 1 is mixed for malignant and benign classes and it is very hard to classify the records using this feature. On the other hand, “area_worst” in Figure 2 is linearly separable and holds the potential for classifying the records. Because of these analyses, this study performed experiments with a varying number of features, and several feature selection methods were added to the experiments to select the best features for classification.

### 3.2. Feature Selection Techniques

Feature engineering is the process of extracting useful features from the raw data to boost the machine learning models’ performance [22,23]. The used dataset contained 10 attributes with each attribute having 3 features, yielding 30 features in total. Such features comprised both primary and derived features. The original dataset contained the values for the given 10 features alone while the mean, standard error, and max constituted the derived features. It was obvious that all features were not good to train the classifiers and important features needed to be selected.

For this purpose, three well-known feature selection approaches were used in this study including principal component analysis (PCA), singular value decomposition (SVD), and the Chi-square (Chi2) method.

#### 3.2.1. Principal Component Analysis

The principal component analysis is a feature selection technique that selects a subset of features that are more useful compared to all features in a dataset. A PCA selects the best features measured using the percentage of consensus in a generalized Procrustes analysis [24,25]. A PCA is used to find the important features based on the covariance matrix [26] of the dataset which increases the performance of machine learning models. It is used to resolve the curse of dimensionality among data with linear relationships. The process of obtaining principal components from the raw dataset is done using

(1) $$C{}_{ij}=\frac{1}{n-1}\sum_{m=1}^{n}\left(X_{im}-\bar{X{}_{i}}\right)\left(X_{jm}-\bar{X{}_{j}}\right)$$

where $C_{ij}$ is the covariance of variable *i* and *j*, and ∑ shows the sum of all objects. $X_{im}$ is the value of variable *i* in object *m*, *i*. $X_{jm}$ is the value of variable *j* in object *m* and $\bar{X_{i}}$, $\bar{X_{j}}$ shows their mean.

#### 3.2.2. Singular Value Decomposition

A singular value decomposition is often called matrix factorization [27] because it is extensively used for matrix decomposition. It is commonly used in a wide array of applications including data reduction, denoising, and compressing [28]. The SVD for the data matrix X(m/n) can be factorized as

(2) $$X=U*D*V^{T}$$

where *U* and *V* represent orthogonal matrices with orthonormal eigenvectors extracted from $XX^{T}$ and $X^{T}X$, respectively. The *D* is a diagonal matrix with *r* elements equal to the root of the positive eigenvalues of $XX^{T}$ or $X^{T}X$. It is represented as $diag(D_{1},D_{2},D_{3},\ldots ,D_{n})$ with singular vectors $D_{1}>D_{2}>D_{3},\ldots ,D_{n}$.

#### 3.2.3. Chi-Square

The Chi-square feature selection technique is used to select the best features which are highly dependent on the correlation between independent variables. When two features are independent, the observed count is close to the expected count and the Chi2 value is small. Thus, a high Chi2 value indicates that the hypothesis of independence is incorrect. In other words, Chi2 is a statistical method used to determine the goodness of fit (GOF) which refers to how close the observed are to those predicted from a hypothesis [29]. The calculation of the Chi2 statistic is quite straightforward and intuitive:

(3) $$x^{2}=\sum \frac{{\left(f_{0}-f_{c}\right)}^{2}}{f_{e}}$$

where $f_{o}$ is the observed frequency and $f_{e}$ is the expected frequency if no relationship existed between the variables.

### 3.3. Supervised Machine Learning Algorithms

Machine learning applications in different domains such as image processing [30], computer vision [31,32], health care [33], edge computing [34], the Internet of things (IoT) [35], etc., helping to make this world fully automated and smart. This study used supervised machine learning models for the automatic detection of breast cancer using FNA features. Five machine learning algorithms were selected for the experiments including RF, SVM, GBM, LG, and KNN. These algorithms were refined using hyperparameter tuning and the list of hyperparameters and their values used for the experiments are given in Table 4.

#### 3.3.1. Random Forest

A random forest is an ensemble model that uses several weak learners (decision trees) to make a final prediction [36]. An RF consists of many decision trees to predict a new instance, where each decision tree provides a prediction for the input data. An RF gathers the predictions and chooses the most voted prediction as the final result. During the tree generation, an RF searches for the best feature among the random subset of features [37]. This results in a higher tree diversity which trades a higher bias for a lower variance, generally yielding an overall better model. An RF can be defined as

(4) $$rf\;=\;modetree_{1},tree_{2},tree_{3},\ldots ,tree_{n}$$

(5) $$rf\;=\;mode\sum_{i=0}^{N}tree_{i}$$

where $tree_{1},tree_{2},tree_{3},\ldots ,tree_{n}$ are trees in the RF and *N* is the number of decision trees. Several parameters are set for the RF to achieve refined results. For example, 100 is commonly used as the n_estimators parameter, which represents the number of decision trees the RF will generate. Similarly, 13 is commonly used as the max_depth parameter, which defines the maximum depth to which each decision can grow. This parameter helps to reduce the complexity of the decision tree, which is useful to avoid overfitting the model.

#### 3.3.2. Support Vector Machine

A support vector machine is a supervised learning algorithm that can be used for classification and regression problems. It is represented as support vector classification (SVC) and support vector regression (SVR). It is used for smaller datasets as it requires a longer processing time. It also tries to maximize the margin between the training data and the classification boundary [38]. SVMs can be trained using stochastic gradient descent (SGD) [39] which is defined as

(6) $$\frac{dA}{d\beta }=\sum_{i=1}^{N}\left\{\begin{array}{cc} if(p^{i}y^{i}<1) & y^{i}X^{i}\\ else & 0 \end{array}\right.$$

where the expression $p^{i}y^{i}<1$ tests whether the point $X^{i}$ is nearer than the margin, and if so, it adds it with sign $y^{i}$. This forces the model to push it further out next time and ignore other points. This SGD training method is much faster than the previous methods and competitive with LR. It is also capable of training in less than one pass over a dataset.

#### 3.3.3. Gradient Boosting Machine

The gradient boosting machine first introduced by Friedman in 2001 is also known as multiple additive regression trees (MART) and gradient boosted regression trees (GBRT) [40,41]. Training using a GBM is sequential, gradual, and additive. In comparison to AdaBoost, which identifies the shortcoming of weak learners using high-weight data points, the GBM does the same by the loss function [42]. The loss function is defined as [43].

(7) Y=ax+b+e

where *e* represents the error and shows the inexplicable data.

The loss function also indicates the fitting of underlying data showing how good the model’s features are. One motivation for using gradient boosting is that it allows for the optimization of user-specified cost functions rather than loss functions. The loss function usually offers less control and has been regarded as unreliable for real-world applications. Three hyperparameters are tuned for the GBM including n_estimators as 100, max_depth parameters as 13, and learning_rate parameter as 0.2 to optimize the good fit of the model.

#### 3.3.4. Logistic Regression (LR)

Logistic regression is one of the most widely used general-purpose models for both classification and regression. LR is used for several problems such as spam filtering, news message classification, website classification, product classification, and classification problems with large and sparse feature sets [44,45,46]. The only problem with the LR is that it can overfit very sparse data, so it is often used with regularization. LR maps the regression value −Xβin(−⋈,⋈) to the range [0, 1] using a logistic function as

(8) $$p\left(X\right)=\frac{1}{1+exp(-X\beta )}$$

The logistic function maps any value on the real line to a probability range i.e., [0, 1]. LR is a generalization of naïve Bayes with binary features. LR can model a naïve Bayes classifier when the binary features are independent. Bayes’s rule for two classes *c* and *d* can be defined as

(9) $$Pr\left(cX\right)=\frac{Pr(Xc)Pr(c)}{Pr(X)}$$

(10) $$=\frac{Pr(Xc)Pr(c)}{Pr(XC)Pr(c)+Pr(Xd)Pr(d)}$$

#### 3.3.5. K-Nearest Neighbors

The k-nearest neighbors algorithm is a technique for classifying objects based on the closest training samples in the problem space. KNN is a type of instance-based learning or lazy learning where the function is only approximated locally, and all computations are deferred until classification [47]. The k-nearest neighbors algorithm is among the simplest of all machine learning algorithms, where an object is classified by a majority vote of its neighbors. The object is assigned to the class which is most common among its k nearest neighbors (k is a positive integer, typically small). If k =1, the object is simply assigned to the class of its nearest neighbor. The KNN algorithm can also be adapted for estimating continuous variables. One such implementation uses an inverse distance weighted average of the k-nearest multivariate neighbors. Research in [48] indicated that the performance of the KNN method did not vary with the size of the target variable but with the type of data. Additionally, a KNN classifier has proved to perform fairly well on smaller datasets such as the Iris flower dataset where 3 classes are defined.

### 3.4. Evaluation Measure

Evaluation measures are used to evaluate the performance of a model for its accuracy and preciseness. Several measures have been presented over the years for classifiers but the accuracy, precision, recall, and F1 measures are among the most commonly used evaluation measures.

- **Accuracy** indicates how many labels out of the total labels are predicted correctly by a classifier. For example, if the total number of testing examples is 100 for benign and malignant samples and models correctly predict 80 examples out of 100, the accuracy of the model will be 80%. The accuracy can be defined by
  (11) $$\mathrm{Accuracy}=\frac{\mathrm{TP}+\mathrm{TN}}{\mathrm{TP}\;+\;\mathrm{TN}\;+\;\mathrm{FP}\;+\;\mathrm{FN}}$$
  where
  **True positive** (TP): the actual class of the observation is benign and models also predict it as benign.
  **True negative** (TN): the actual class of the observation is malignant and models also predict it as malignant.
  **False positive** (FP): the actual class of the observation is malignant and models predict it incorrectly as benign.
  **False negative** (FN): the actual class of the observation is benign and models predict it incorrectly as malignant.
- **Recall** is also known as sensitivity and can be defined as the ratio of the total number of correctly predicted positive examples to the total number of positive examples. A high value of recall indicates that the class is correctly recognized (a small number of FNs).
  (12) $$\mathrm{Recall}=\frac{\mathrm{TP}}{\mathrm{TP}\;+\;\mathrm{FN}}$$
- **Precision** is also known as the exactness of classifiers. Precision can also be defined as the number of TPs divided by the number of TPs and FPs.
  (13) $$\mathrm{Precision}=\frac{\mathrm{TP}}{\mathrm{TP}\;+\;\mathrm{FP}}$$
- **F1 score** is also known as the F measure and it is the harmonic mean of the precision and recall scores. The F measure will always be nearer to the smaller value of precision or recall. The F1 score can be defined as follows:
  (14) $$F1\;\mathrm{score}=2\times \frac{\mathrm{Precision}\times \mathrm{Recall}}{\mathrm{Precision}\;+\;\mathrm{Recall}}$$

### 3.5. Proposed Methodology

**Figure 3:**
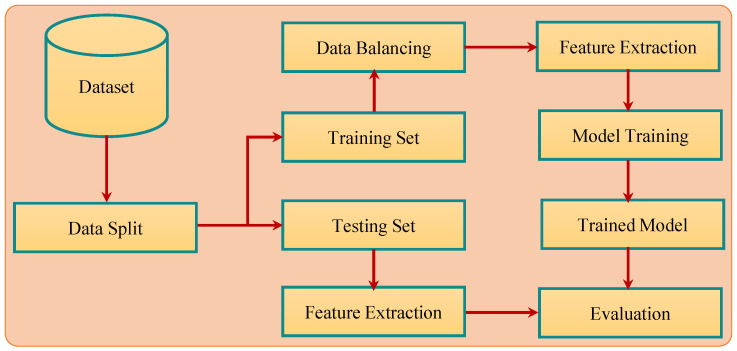
The pipeline of the proposed methodology for breast cancer detection.

Figure 3 shows the pipeline of the proposed methodology for breast cancer detection. Experiments were performed using two different approaches to analyze the impact of data balance on accuracy. As shown in Table 3, the number of records for benign and malignant classes was not equal, which caused a data imbalance and affected the learning process of the classifier. To analyze the impact of data imbalance on classification accuracy, experiments were performed using balanced data with SMOTE upsampling and imbalanced data. For both courses of action, feature selection was performed with PCA, SVD, and Chi2 after splitting the data into training and testing sets with a 75:25 ratio. Machine learning models including SVM, RF, GBM, LR, and KNN were trained on the training data and later, the trained models were evaluated using the testing data.

The performance of the selected classifiers was evaluated from three perspectives. First, the performance using the testing data with the selected performance measures of accuracy, precision, recall, and F1 score was assessed. Secondly the performance evaluation was carried out with different feature sets such as 10 features, 20 features, and 30 features, etc., to find the optimal feature set size for each classifier. In addition, the influence of feature selection using the PCA, Chi2, and SVD feature selection approaches was evaluated. Lastly, the performance of the selected machine learning classifier using the proposed pipeline was compared with several state-of-the-art approaches for analyzing the striking differences in classification accuracy.

## 4. Results and Discussions

Experiments were performed with an imbalanced dataset, as well as a balanced dataset using the SMOTE approach. Results for each scenario are discussed separately.

### 4.1. Results Using Imbalanced Dataset

#### 4.1.1. Performance of Classifiers without Feature Selection

First, experiments were performed with all the features from the data, and the feature importance from PCA, SVD, and Chi2 was not used. It indicated all 30 features from the dataset were used both for training and testing. The same train/test split ratio of 75:25 was used for all experiments. Experimental results are given in Table 5. Results indicated that using all features, the KNN classifier performed the best with an accuracy of 0.94 while the GBM and LR classifiers performed poorly each with an accuracy of 0.91. The performance of the MLP and RF was similar with an accuracy of 0.93.

#### 4.1.2. Performance of Classifiers Using Different Feature Sets

Further experiments were performed using various feature sets where the features were selected using PCA, SVD, and Chi2. The number of features varied from 10 to 30 with a difference of 5 features for each experiment. For example, Table 6 shows the results when the 10 most important features were selected using the PCA, SVD, and Chi2 techniques. Results showed that the accuracy of the RF using 10 features from an SVD was the same as that when using 30 features. The SVD considered less important features for calculating the accuracy while the PCA and Chi2 techniques skipped the less important features. The accuracy of the MLP and KNN classifiers improved with a PCA from 0.93 and 0.94 to 0.95 and 0.95, respectively.

Table 7 shows the results of the machine learning classifiers when the top 15 features were selected. The RF, GBM, LR, and KNN classifiers gave the best results with Chi2 features in comparison to SVD- and PCA-derived features. In the Chi2 technique, due to the absence of association between two cross-tabulated variables, the percentage distributions of the dependent variable within each category of the independent variable are identical, which affects the accuracy of results. The performance of the SVM was the same with 10 and 15 features while the performance of the GBM improved with 15 features. The highest classification accuracy using 15 features was when a KNN classifier was used with Chi2 features and an MLP with either SVD or PCA features.

Further experiments using 20 and 25 features for each feature selection approach indicated that there was no improvement in the classification accuracy with 25 features, so only the results with 20 features are presented in Table 8. The results suggested that the accuracy of the RF and SVM improved with 20 features with Chi2-selected features while the performance of the GBM tended to go down. The LR and MLP performed almost similarly with 15 and 20 features while the performance of the KNN model was enhanced with an SVD but degraded with the Chi2 and PCA feature selection techniques. The highest accuracy of 0.96 using 20 features was obtained from the MLP when used with either of the three feature selection approaches.

**Figure 4:**
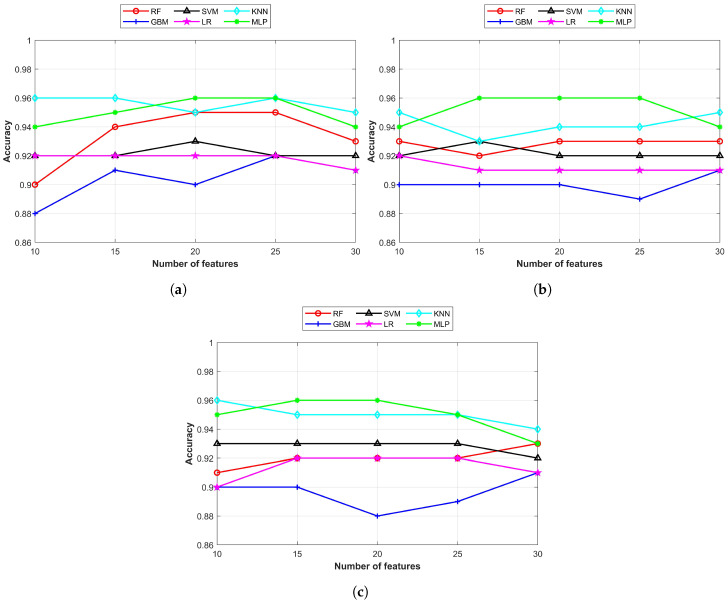
Comparison of classification accuracy using various number of features: (**a**) features using the Chi2, (**b**) SVD, and (**c**) PCA feature selection approaches.

A comparison of the classifiers’ accuracy with each feature selection approach is shown in Figure 4 where the x-axis indicates the number of selected features while the y-axis represents the highest achieved accuracy. Figure 4 shows that the MLP and KNN classifiers consistently showed better performance on all feature selection approaches that other machine learning classifiers. The highest achieved accuracy on the unbalanced dataset was with the MLP and KNN classifiers with different feature sets selected from different feature selection approaches. The highest accuracy of 0.96 for the MLP was using 15, 20, and 25 features from the SVD and 15 and 20 features from the PCA. On the other hand, the KNN classifier achieved the same using 10, 15, and 25 Chi2 features and 10 PCA features.

### 4.2. Experimental Results with Balanced Data

After upsampling, the distribution of the number of samples in each class is given in Table 9. The purpose of upsampling the records for the malignant class was to balance the records so that the classifiers could be properly trained to achieve an increased accuracy. The number of samples used for training plays a major role in the resulting accuracy. Imbalanced datasets where the number of samples for the minor class is low cause the classifier to be insufficiently trained and result in a higher number of inaccurate predictions [49]. Many classifiers implicitly assume that the data are balanced; consequently, the minor class is ignored, and classifiers are biased toward the majority.

#### 4.2.1. Results with All Features

The same procedure was adopted with the balanced dataset as for the imbalanced dataset where the size of the feature set was changed from 10 to 30 gradually, with each of the selected feature selection approaches. initially, all 30 features were selected for the experiments with the machine learning classifiers. Experimental results are given in Table 10. Results indicated that balancing the dataset led to a better classification accuracy from all the classifiers. The accuracy was improved substantially for all classifiers and especially the KNN classifier, which showed an accuracy of 0.99 using all 30 features.

#### 4.2.2. Experimental Results on Balanced Dataset Using Different Feature Sets

In addition to the selection of all 30 features from the balanced dataset, several experiments were performed using 10, 15, 20, and 25 features. These features were selected using the PCA, Chi2, and SVD feature selection approaches. Table 11 and Table 12 show the classification accuracy using 10 and 15, 20 and 25 features, respectively, with the PCA, Chi2, and SVD approaches.

The results indicated that balancing the dataset lead to an increased performance where the breast cancer detection accuracy from all the classifiers was increased significantly. Balancing the dataset mad the number of training samples almost equal for both classes, which increased the learning capability of the classifiers. Hence, the prediction accuracy was improved.

Furthermore, the results suggested that the performance of the models increased when the number of features was reduced. Originally, the dataset contained 10 features while the additional 20 features were derived features. However, all the derived features were not necessarily appropriate to contribute to a better prediction accuracy. The same could be said for the original 10 features; thus, feature selection was an important process whereby a higher accuracy could be achieved using less features. For this purpose, this study utilized the PCA, Chi2, and SVD approaches. The results given in Table 11 and Table 12 indicate that the KNN method outperformed all other classifiers by achieving an accuracy of 100% for two feature set sizes, 15 features and 20 features. It achieved an accuracy of 100% when trained on the most important 15 features selected using the PCA algorithm. However, its performance was superior when trained on the most important 20 features as it achieved an accuracy of 99.0% with PCA and Chi2 features each and 100% with SVD features.

**Figure 5:**
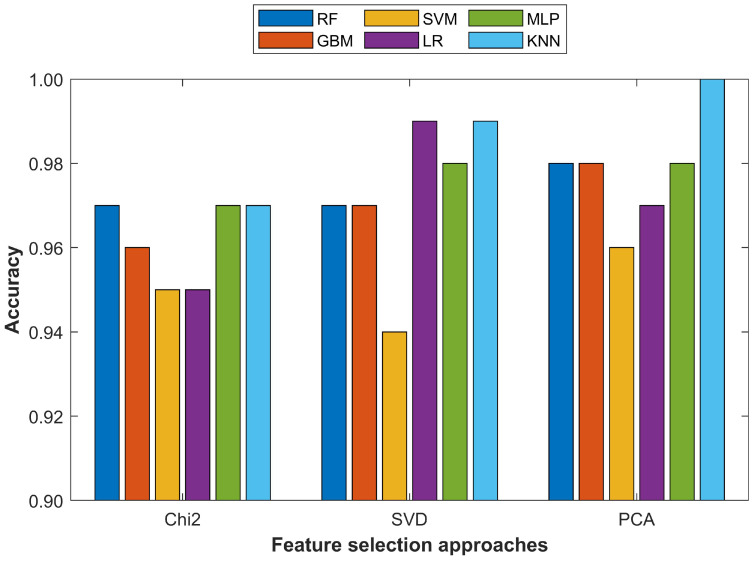
Comparison of different machine learning models with 15 selected features from Chi2, SVD, and PCA approaches.

**Figure 6:**
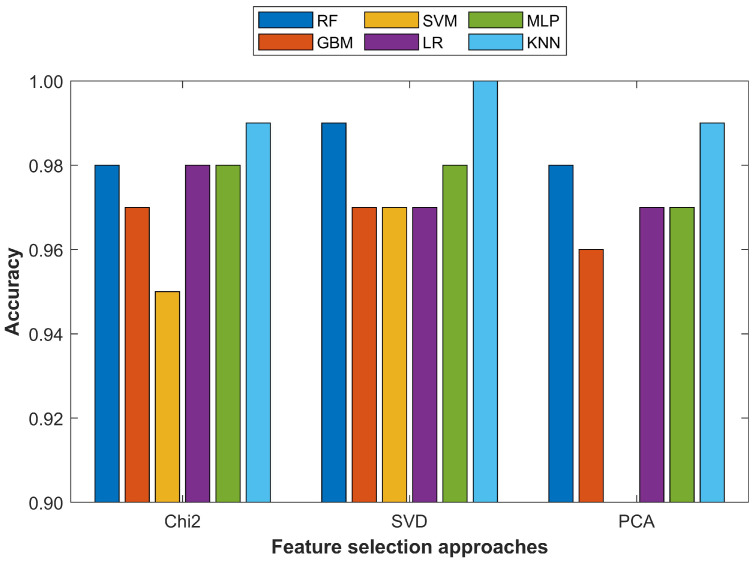
Comparison of different machine learning models with 20 selected features from Chi2, SVD, and PCA approaches.

For better understanding the results, the classification accuracy of all the classifiers using the most important 15 and 20 features from the PCA, Chi2, and SVD techniques is shown in Figure 5 and Figure 6, respectively.

### 4.3. Results Using K-Fold Cross-Validation

In this section, we present the results of a k-fold cross-validation using all feature engineering techniques. We selected the best 15 results and deployed the machine learning models. We used 10 folds to perform the experiments. The results of the models are presented in Table 13. The experimental results revealed that the models also performed better using the k-fold validation approach, similar to the train–test–split approach. The SVM and LR showed significant results using PCA features as the SVM and LR both achieved a mean accuracy of 0.98 with a ±0.02 standard deviation. Similarly, with Chi2 features, the RF, SVM, and LR achieved a 0.97 mean accuracy score. These results showed that the proposed approach was not overfitting because the 10-fold accuracy was still high at 0.98 with a low standard deviation of ±0.02.

### 4.4. Discussion

**Figure 7:**
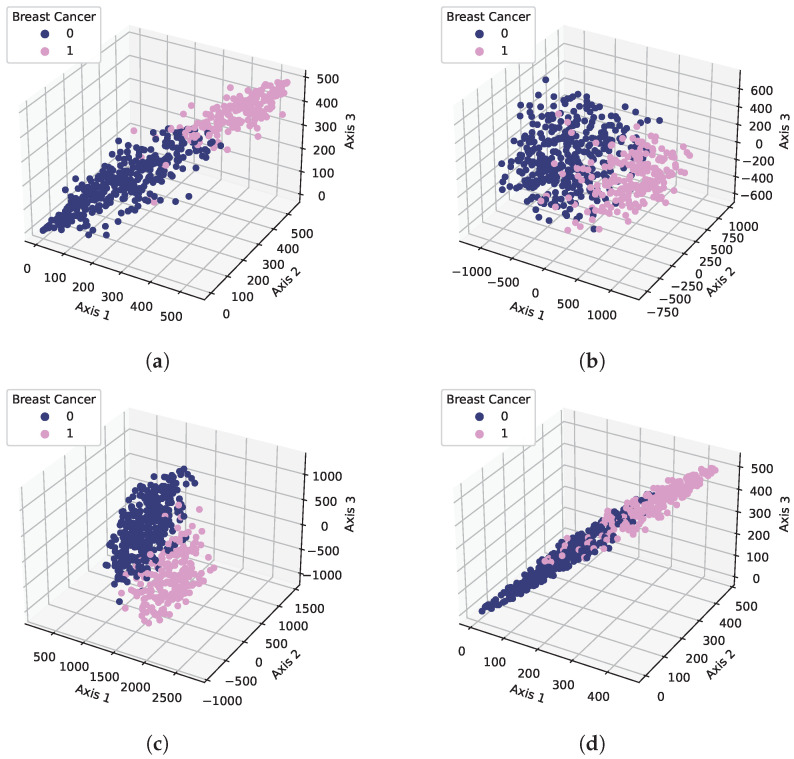
Feature space visualization using feature selection techniques: (**a**) Chi2, (**b**) PCA, (**c**) SVD, and (**d**) original.

Our results showed that the models’ performance varied with the change in feature selection techniques and the number of features used for the experiments. The underlying reason was the impact of the feature space on the models’ learning process. When we selected the best features, the feature space became more linearly separable which helped to improve the performance of the machine learning models. For clarification, the feature space of the used dataset is shown in Figure 7. For this purpose, a scatter plot was used to show the feature space. We reduced the dataset dimension with the PCA, Chi2, and SVD techniques into three dimensions and then illustrated it on a scatter plot while in the original dataset case, we used three random features and visualized the feature space. We can see that in the case of the used feature selection techniques, there were only a few samples that were overlapping but in the original dataset case, the overlapped sample count was higher which led to poor learning and low accuracy.

For analyzing the efficacy of the proposed pipeline and adopted strategy for breast cancer detection using FNA features, its performance was compared with several state-of-the-art approaches. Table 14 shows the comparison between the proposed strategy and previous works on breast cancer detection. Comparison results indicate that the proposed methodology outperformed state-of-the-art approaches and achieved an accuracy of 100%. This accuracy was achieved using the KNN approach with the 15 most important FNA features which were selected using the PCA algorithm. It proved that selective features played a more important role to enhance the prediction performance than using a large set of features that were not prioritized with a feature selection approach. Similarly, all derived features do not contribute to elevating the performance of a classifier, and the selection of important features that carry a higher importance can play an important part to increase the accuracy of machine learning classifiers. Most of the studies used the WBCD dataset, which is an imbalanced dataset; we applied SMOTE for data balancing. We also applied feature selection techniques that selected the best features for model training; the number of features could be 30, 20 or 15 features. Despite obtaining better results from the models, several limitations still exist in this study. The size of the dataset was small and the dataset was imbalanced as well. By improving these limitations in the future, more accurate results can be obtained.

## 5. Conclusions

This study proposed a methodology for breast cancer detection using fine-needle aspiration features. Experiments were conducted with a threefold purpose. First, the impact of the imbalanced data size was analyzed on the classification accuracy of six classifiers including RF, SVM, GBM, LR, MLP, and KNN. For this purpose, the dataset was upsampled for the minor class using the SMOTE approach. Secondly, the influence of the feature set size was analyzed using various feature sets with selected machine learning classifiers with all and selected features, respectively. Important features were selected using three feature selection approaches: PCA, Chi2, and SVD. Thirdly, analyses were performed to validate the effect of the primary and derived features on the classification accuracy. The results indicated that an imbalanced dataset led the classifiers to a biased attitude toward the minor class and produced incorrect predictions, which reduced the classification performance. Balancing the dataset with SMOTE increased the performance of all the classifiers and KNN especially. Changing the feature set size was also important and an increase in the feature set size tended to degrade the performance of the classifier. It showed that increasing the feature set did not necessarily improve the performance, especially when the feature vector contained derived features. The results suggested that the derived features did not guarantee enhanced performance unless they were prioritized concerning their importance using a feature selection approach. The proposed methodology provided a 100% breast cancer prediction accuracy with the KNN approach using the 15 most important features selected from the PCA algorithm and outperformed state-of-the-art approaches. The performance of the KNN was superior when used with the 20 most important features as it reached 99.0% with the PCA and Chi2 techniques each and 100% when an SVD was used. The proposed approach was limited by the fact that experiments were performed on the WBCD, one of the most widely used datasets for breast cancer detection and did not guarantee the same results on other datasets. We intend to use more datasets to generalize the results.

## Figures and Tables

**Table 1:** Summary of the discussed research works.

Ref.	Year	Model/Techniques	Dataset
[15]	2007	ANN	WPBC and Love data
[14]	2010	SOM, RBF, GRNN, PNN, PCA	WBCD and NHBCD
[12]	2012	C4.5, C5, CART, CHAID, SLIQ, SPRINT, ScalParc	WBCD
[10]	2013	MLP, SVM, RVM	WBCD
[16]	2014	SVM, naïve Bayes, ANN	WBCD
[13]	2017	SMO, IBK, BF	WBCD
[17]	2017	Fuzzy artificial immune system, k-nearest neighbors, 10-fold cross-validation	WBCD

**Table 2:** List of attributes and their measured values after the fine-needle inspiration process.

Attribute Name	Attribute Description	b	St. Error	Max
Radius	Mean distances from the center to the points on the perimeter	6.97–28.12	0.11–2.8	7.9–36.4
Texture	Standard deviation of grayscale values	9.72–39.27	0.36–4.8	12–49.5
Perimeter	Real value	43.79–188.5	0.7–21.9	50–251
Area	Real value	143.5–2501	6.8–542	185–424
Smoothness	Local variation (in radius lengths)	0.053–0.163	0.0–0.03	0.0–0.22
Compactness	Formula to compute: (perimeter${}^{2}$/area-1.0)	0.019–0.345	0.00–0.1	0.02–12
Concavity	The severity of concave portions of the contour	0.000–0.427	0.0–0.39	0.0–1.05
Concave points	Number of concave portions of the contour	0.000–0.201	0.0–0.05	0.0–0.29
Symmetry	Real value	0.106–0.304	0.0–0.07	0.15–0.6
Fractal dimension	(“coastline approximation”-1)	0.050–0.097	0.0–0.03	0.05–0.2

**Table 3:** Data count for both classes in the dataset.

Data	Training	Testing	Total
Benign	259	98	357
Malignant	167	45	212
Total	426	143	569

**Table 4:** Hyperparameters and associate values used for experiments.

Model	Hyperparameters
Random forest	n_estimators = 100, max_depth = 50
Support vector machine	kernel = linear, C = 3.0
Gradient boosting	n_estimators = 100, max_depth = 50, learning_rate = 0.2
Logistic regression	solver = liblinear, C = 3.0

**Table 5:** Classification results using all 30 features from the dataset.

Classifier	Accuracy
RF	0.93
SVM	0.92
GBM	0.91
LR	0.91
MLP	0.93
KNN	0.94

**Table 6:** Performance of classifiers with 10 most important features.

Model	Chi2	SVD	PCA
RF	0.90	0.93	0.91
SVM	0.92	0.93	0.93
GBM	0.88	0.90	0.90
LR	0.92	0.92	0.90
MLP	0.94	0.94	0.95
KNN	0.95	0.95	0.95

**Table 7:** Classification accuracy of machine learning classifiers using 15 most important features.

Model	Chi2	SVD	PCA
RF	0.94	0.92	0.92
SVM	0.92	0.93	0.93
GBM	0.91	0.90	0.90
LR	0.92	0.91	0.92
MLP	0.95	0.96	0.96
KNN	0.96	0.93	0.95

**Table 8:** Accuracy of classifiers using 20 most important features.

Model	Chi2	SVD	PCA
RF	0.95	0.93	0.92
SVM	0.93	0.92	0.93
GBM	0.90	0.90	0.88
LR	0.92	0.91	0.92
MLP	0.96	0.96	0.96
KNN	0.95	0.94	0.95

**Table 9:** Number of samples for each class after applying SMOTE.

	Benign	Malignant	Total
Training set	271	264	535
Testing set	93	86	179
Total	364	350	714

**Table 10:** Experiment results using all 30 features from the balanced dataset.

Classifier	Accuracy
RF	0.98
SVM	0.97
GBM	0.95
LR	0.98
MLP	0.95
KNN	0.99

**Table 11:** Performance of classifiers with 10 and 15 most important features from a balanced dataset.

Model	10 Features			15 Features		
	Chi2	SVD	PCA	Chi2	SVD	PCA
RF	0.96	0.97	0.98	0.97	0.97	0.98
SVM	0.95	0.98	0.97	0.96	0.97	0.98
GBM	0.95	0.98	0.97	0.95	0.94	0.96
LR	0.96	0.98	0.97	0.95	0.99	0.97
MLP	0.98	0.97	0.98	0.97	0.98	0.98
KNN	0.98	0.98	0.99	0.97	0.99	1.00

**Table 12:** Performance of classifiers with 20 and 25 most important features from a balanced dataset.

Model	20 Features			25 Features		
	Chi2	SVD	PCA	Chi2	SVD	PCA
RF	0.98	0.99	0.98	0.98	0.98	0.99
SVM	0.97	0.97	0.96	0.98	0.96	0.96
GBM	0.95	0.97	0.96	0.97	0.98	0.96
LR	0.98	0.97	0.97	0.96	0.96	0.96
MLP	0.98	0.98	0.97	0.97	0.97	0.97
KNN	0.99	1.00	0.99	0.99	0.99	0.99

**Table 13:** k-Fold cross-validation by all feature selection techniques.

Models	Actual Features	PCA Features	SVD Features	Chi2 Features
RF	0.94 (±0.04)	0.97 (±0.03)	0.96 (±0.03)	0.97 (±0.04)
SVM	0.94 (±0.03)	0.98 (±0.02)	0.97 (±0.04)	0.97 (±0.03)
GBM	0.93 (±0.04)	0.93 (±0.03)	0.94 (±0.03)	0.94 (±0.03)
LR	0.94 (±0.03)	0.98 (±0.02)	0.96 (±0.02)	0.97 (±0.02)
KNN	0.94 (±0.03)	0.97 (±0.03)	0.96 (±0.02)	0.96 (±0.02)

**Table 14:** Comparison of classification accuracy with state-of-the-art approaches for breast cancer detection.

Ref.	Models	Features	Cross-Validation	Dataset	Accuracy
[18]	GONN	9	10 folds	WBCD	98.24%, 99.63% and 100% for 50–50, 60–40, 70–30 train–test split, respectively
[13]	SMO	10	No	WBCD	96.2%
[10]	SVM, MLP	N/M	No	WBCD	99%, 99.28%
[19]	AR, NN	9	3 folds	WBCD	95.6%
[16]	SVM	30	No	WDBC + WPBC	97.0%
[17]	K-NN	9	10 folds	WBCD	99.14%
Proposed	KNN	15	Yes	WBCD	100%

## Data Availability

Not applicable.
